# Supplementary material for: Frizzled-8 integrates Wnt-11 and transforming growth factor-β signaling in prostate cancer
Source: Nat Commun. 2018 May 1;9:1747. doi: 10.1038/s41467-018-04042-w (PMC5931552; doi:10.1038/s41467-018-04042-w)

Frizzled-8 integrates Wnt-11 and Transforming Growth Factor-β Signaling in Prostate Cancer

Murillo-Garzón et al.

Supplementary Table 1

Distributor	siRNA/dsiRNA	SEQUENCE 5´→ 3´	Concentration (nM)
SMARTpool (Dharmacon, Thermo Fisher)	siFZD2	CCACGUACUUGGUAGACAU	25
		GAACUGCGCUUCUUCCUGU	
		GGAGGAAGUUCUACACUCG	
		GCUACAAGUUUCUGGGCGA	
SMARTpool (Dharmacon, Thermo Fisher)	siFZD3	CCAAAUACUCCUAUCAUAA	25
		ACAGAUCACUCCAGGCAUA	
		GUUCGAAGCUCAUGGAGAU	
		UGAUUGAUGUCACAAGAUU	
SMARTpool (Dharmacon, Thermo Fisher)	siFZD4	GAUCGAUUCUUCUAGGUUU	25
		UCACACCGCUCAUCCAGUA	
		GGACAAAGACAGACAAGUU	
		CCAAGGAGUUCACUGAUAU	
SMARTpool (Dharmacon, Thermo Fisher)	siFZD5	GCAUUGUGGUGGCCUGCUA	50
		GCACAUGCCCAACCAGUUC	
		AAAUCACGGUGCCCAUGUG	
		GAUCCGCAUCGGCAUCUUC	
SMARTpool (Dharmacon, Thermo Fisher)	siFZD8	AGACAGGCCAGAUCGCUAA	25
		ACACCUACAUGCCCAAUCA	
		UCACCGUGCCGCUGUGUAA	
		CGGCGAGCUCCGUGUCUUA	
Integrated DNA Technologies (IDT)	dsiFZD8	rArGrCrUrCrCrGrUrGrUrCrUrUrArUrCrCrArArArGrCrAGA	25
		rUrCrUrGrCrUrUrUrGrGrArUrArArGrArCrArCrGrGrArGrCrUrCG	

List of siRNAs and dsiRNAs used in this study. Distributors, sequences, and concentrations are indicated.

Supplementary Table 2

PRIMER	SEQUENCE 5→3´	PRIMER CONCENTRATION (nM)	PRIMER	SEQUENCE 5→3´	PRIMER CONCENTRATION (nM)
FZD1 F	CATTCTCTAGTGTCTAAAACCT	300	LRP6 F	TGAGGGTCTGCGTGAAATCC	300
FZD1 R	TAACATTTTATAACCCATATCTTC	300	LRP6 R	TGGGAACAACCCCCATTGTC	300
FZD2 F	ATTCGCTGCACCAAGTGCTTC	300	GPC4 F	TCGTGACTGTGAAGCCATGT	300
FZD2 R	CGAATGCTTCCCCTAGACATGA	300	GPC4 R	TCTAGCCTCTCTGCCACCAT	300
FZD3 F	TGATGGCTCTCATAGTTGGCA	300	MuSK F	CCCACCATCACCTGGATTGAA	300
FZD3 R	ACCTGTCGGCTCTCATTCAC	300	MuSK R	TGTGTAGAGTCCTGGCTTGG	300
FZD4 F	GACAACTTTCACACCGCTCA	900	WNT11 F	AGACCGGCGTGTGCTATG	900
FZD4 R	TCTTCTCTGTGCACATTGGC	900	WNT11 R	CACCTGTGCAGACACCAGAC	900
FZD5 F	GTACCCAGCCTGTCGCTAAA	300	ATF2 F	AGTCCTTTACCTCACCCAGAGT	300
FZD5 R	CCGAGAAGAGCAGACAGTCC	300	ATF2 R	GATGTGGGCTGTGCAGTTTG	300
FZD6 F	TCCCAGATGTATGAAAATGGC	900	JUN F	TGAGTGACCGCGACTTTTCA	300
FZD6 R	CCAGATTTGCGAGAGGAAGA	900	JUN R	TTAAGATGCCTCCCGCACTC	300
FZD7 F	GCCTCTGTTCGTCTACCTC	300	CREB F	AGCCCAGCCACAGATTGCCAC	300
FZD7 R	GTCGTGTTTCATGATGGTGC	300	CREB R	GTTACGGTGGGAGCAGATGAT	300
FZD8 F	GCTCTACAACCGCGTCAAGA	900	AXIN2 F	AAGTGCAAACTTTCGCCAAC	300
FZD8 R	GCTGAAAAAGGGGTTGTGGC	900	AXIN2 R	ACAGGATCGCTCCTCTTGAA	300
FZD9 F	TGGCGGTCTTCATGCTCAAA	300	CDH1 F	AGCAGAACTAACACACGGGG	600
FZD9 R	TATCTTGCGGTAGCACAGGC	300	CDH1 R	ACCCACCTCTAAGGCCATCT	600
FZD10 F	CCGCCTCACACCCCAGTGGAT	300	CLDN1 F	CTGTCATTGGGGGTGCGATA	600
FZD10 R	TTCTGTCTTGCAGAGAGACTATT	300	CLDN1 R	CTGGCATTGACTGGGGTCAT	600
VANGL1 F	GCTTCTACAGCCTGGGACAC	300	CDH2 F	CATGAAGGACAGCCTCTTCTCAA	300
VANGL1 R	GCTCGGAATTTGGAGGCTGT	300	CDH2 R	GCTTCTCACGGCATACACCAT	300
VANGL2 F	AAGTCGGGCCACTCCCGCAG	300	VIM F	GCTTCAGAGAGAGGAAGCCG	600
VANGL2 R	CCCATCTCGACTCTTAGAGC	300	VIM R	AAGGTCAAGACGTGCCAGAG	600
ROR1 F	AAAGAGCTACCTCTTTCTGCTGTACG	300	SNAI F	CCCTGGCTGCTACAAGGC	600
ROR1 R	CTTCTTGTTGAAATTCCGTCCATTG	900	SNAI R	TGAGTGGGTCTGGAGGTGG	600
ROR2 F	GGCAGAACCCATCCTCGTG	300	SNAI2 F	GCCAAACTACAGCGAACTGG	300
ROR2 R	CGACTGCGAATCCAGGACC	300	SNAI2 R	AGTGATGGGGCTGTATGCTC	300
RYK F	TCTCTACCTGAGCGAGGACG	300	TWIST1 F	GCCACTGAAAGGAAAGGCATC	600
RYK R	CCAGGTGAAGTGCAGGAAAT	300	TWIST1 R	TGGTTTTGCAGGCCAGTTTG	600
PTK7 F	GTTGCCTCTGCTCAGCGT	300	ZEB1 F	AAGAATTCACAGTGGAGAGAAGCCA	300
PTK7 R	CCCTGCAGTGCATCCTG	300	ZEB1 R	CGTTTCTTGCAGTTTGGGCATT	300
LGR4 F	GGCCTTGTCTGGGTTGAAAG	300	MMP9 F	TTGGTCCACCTGGTTCAACT	300
LGR4 R	CAAAGCACTCAGCCCTCGAA	300	MMP9 R	TCAACTTGGTCCACCTGGTT	300
LGR5 F	GGGAGCATTCACTGGCCTTTA	300	PAI1 F	CAATCGCAAGGCACCTCTGA	600
LGR5 R	TCCAGACGCAGGGATTGAAG	300	PAI1 R	TTCACCAAAGACAAGGGCCA	600
LRP4 F	GGTCTTGGTCAGCCATGTGT	300	36B4 F	GTGTTCGACAATGGCAGCAT	300
LRP4 R	ACACGCTGGATTGACTTGGT	300	36B4 R	AGACACTGGCAACATTGCGGA	300
LRP5 F	AAGAGGAAGGAGATCCTGAGTG	300			
LRP5 R	ATTGTCCTCCTCACAGCGAGT	300			

List of primers used in this study, with their sequences and concentration.

Supplementary Table 3

Antibody	Company	Catalog Number	Species	Application	Concentration
**pSMAD2**	Cell Signaling	138D4	Rabbit	WB	1:1000
**pSMAD3**	Abcam	ab52903	Rabbit	WB	1:2000
**SMAD2/3**	Santa Cruz	sc-133098	Rabbit	WB	1:1000
**PA tag**	Novus Biological	NBP-03952	Rat	IP	1 ug
				WB	1:1000
**1D4**	Santa Cruz	sc-57432	Mouse	IP	1 ug
				WB	1:2000
				IF	1:200
**HA**	Santa Cruz	sc-805	Rabbit	WB	1:1000
				IF	1:200
**Flag**	Sigma-Aldrich	F1804	Mouse	WB	1:1000
				IF	1:200
**FZD8**	LS Bio	LS-C120599	Rabbit	WB	1:2000
				IHC	1:100
**FZD8**	Aviva Systems Biology	OAEB02431	Goat	IF	1:50
**IgG**	GenScript	A00166	Human	WB	1:5000
**Wnt-11**	R&D	AF2647	Goat	WB	1:2000
				IF	1:50
				IHC	1:100
**N-cadherin**	Santa Cruz	sc-393933	Mouse	WB	1:500
**Vimentin**	Santa Cruz	sc-373717	Mouse	WB	1:500
**Vimentin**	Novocastra Leica Biosystems	NCL-L-VIM-V9	Mouse	IHC	1:1000
**Claudin-1**	Santa Cruz	Sc-166338	Mouse	WB	1:500
**Pan-Citokeratin**	Thermo Scientiific	MA5-13156(AE1/AE3)	Mouse	IHC	1:200
**β-tubulin**	Sigma-Aldrich	T5293	Mouse	WB	1:5000
**GAPDH**	SIGMA	G8795	Mouse	WB	1:2000

List of antibodies used in this study, with their companies, catalog numbers, species and concentrations for each application; WB western blotting, IP immunoprecipitation, IF immunofluorescence, IHC immunohistochemistry.

Supplementary Table 4

Parameter	Explanation	Unit
**Area**	Area of the segmented structure	pixels
**Roundness**	Roundness of the segmented structure	%
**Roughness**	Roughness of the surface of the segmented structure	%
**AppIndex**	Indexes the severity of the invasiveness by utilising Roundness and Roughness parameters	ratio

Definitions of parameters evaluated with AMIDA analysis of 3D culture assays.

Supplementary Table 5

Characteristic	Number of patients
Gleason (patients)	
3 + 3	6
3 + 4	59
4 + 3	27
4 + 4	0
4 + 5	7
Stage	
pT2a	1
pT2b	1
pT2c	48
pT3a	29
pT3b	11
Lymph node metastasis	
Yes	1
No	89
Perineural invasion	
Yes	37
No	53
Lymphovascular invasion	
Yes	7
No	83
Inflammation	
Yes	27
No	72
Gleason (sections analyzed)	
3 + 3	47
3 + 4	21
4 + 3	14
4 + 4	7
4 + 5	1
No cancer	9

Clinical and pathological characteristics of the prostate cancer patients used in the TMAs.

Supplementary Table 6

	Ct C4-2B	Ct PC-3M	Ct VCaP
FZD1	UND	UND	UND
FZD2	26		
FZD3	23		
FZD4	21		
FZD5	21		
FZD6	22		
FZD7	25		
FZD8	26		
FZD9	26		
FZD10	UND	UND	27
VANGL1	21		
VANGL2	28		
ROR1	24		
ROR2	30		
RYK	20		
PTK7	25		
LGR4	21		
LGR5	UND	27	
LRP4	31		
LRP5	20		
LRP6	21		
GPC4	23		
MuSK	UND	28	
WNT11	30		

Q-PCR Ct values for listed genes in C4-2B cells used for normalization in Supplementary Fig. 1; PC-3M or VCaP were used for normalization in cases where a gene was undetectable (UND) in C4-2B cells.

Supplementary Table 7

	Cancer vs benign		C.stroma vs B.stroma	
**Correlation**	**FZD_8**	**Wnt-11**	**FZD_8**	**Wnt-11**
**Chi Pearson**	<0.001	<0.001	<0.001	<0.001
**Fisher Exact Two tails**	1.13E-11	4.56E-09	7.27E-15	4.68E-12

Comparison of FZD8 and Wnt-11 protein expression in benign and malignant prostate tissue. FZD8 and Wnt-11 protein expression levels were compared in tumor and benign epithelium and cancer stroma and benign stroma. Both were significantly higher in cancer and significantly lower in cancer stroma. Pearson Chi-square test with correction and Fisher´s exact test, two-sided are shown

Supplementary Table 8

Antigen Staining score	Category n (%)	Category n (%)	Chi-Square; Fisher´s Exact
**Fzd-8**			
	Benign	Cancer	<0,001; 1,13E-11
Low	69 (76,7)	22 (25,6)	
High	21(23,3)	64 (74,4)	
	Gleason ≤ 3,4	Gleason ≥4,3	NS
Low	47 (52,8)	19 (12,3)	
High	21 (23,6)	2 (2,24)	
	pT2	pT3	NS
Low	16 (17,9)	7 (7,8)	
High	33 (37,1)	33 (37,1)	
	PNI+	PNI-	NS
Low	7 (7,8)	16 (17,9)	
High	28 (31,4)	38 (42,7)	
**Wnt-11**			
	Benign	Cancer	<0,001; 4,56E-9
Low	75 (83,3)	35 (40,7)	
High	15 (16,6)	51 (58,6)	
	Gleason ≤ 3,4	Gleason ≥4,3	NS
Low	36 (41,3)	15 (17,2)	
High	31 (35,6)	5(5,7)	
	pT2	pT3	NS
Low	23 (26,4)	13 (14,9)	
High	25 (28,7)	26 (29,8)	
	PNI+	PNI-	NS
Low	12 (13,8)	24 (27,6)	
High	28 (32,2	23 (26,4)	
**Fzd-8 stroma**			
	Benign	Cancer	<0,001; 7,27E-15
Low	14 (15,5)	63 (72,4)	
High	76 (84,4)	24 (27,6)	
	Gleason ≤ 3,4	Gleason ≥4,3	NS
Low	22 (24,7)	2 (2,2)	
High	46 (51,7)	19 (21,3)	
**Wnt-11 stroma**			
	Benign	Cancer	<0,001; 4,68E-12
Low	13 (14,4)	56 (64,4)	
High	77 (85,5)	31 (35,6)	
	Gleason ≤ 3,4	Gleason ≥4,3	NS
Low	24 (27,3)	7 (7,9)	
High	44 (50)	13 (14,7)	

Statistical analysis of FZD_8 and Wnt-11 staining intensities in TMAs. Adjacent sections of tumor and benign prostate from 99 patients were analyzed; 9 tumor sections were subsequently found to contain no cancer and were removed from the analysis, as were samples stained for Wnt-11 and FZD_8 that were missing/damaged. Staining was classified as low (score 0 or 1) or high (score 2 or 3). Entries are absolute numbers with % in parentheses. Statistical significance was determined using Chi-squared test, Pearson correction and Fisher´s exact test, two-sided; $p < 0.05$ was considered to be statistically significant.

Supplementary Figure 1. Expression of WNT11 and WNT receptors in prostate cancer cell lines

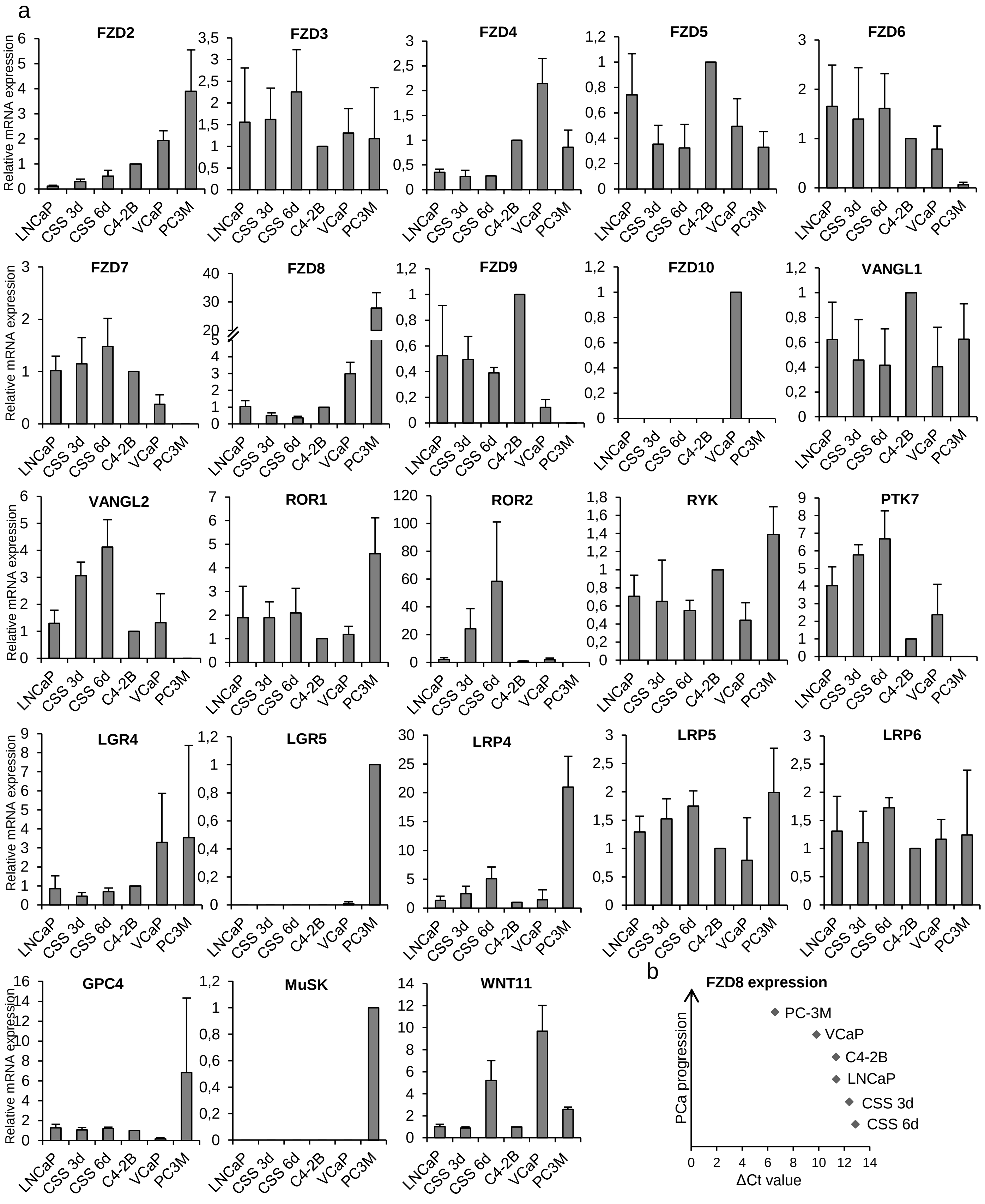

Supplementary Figure 1. Expression of WNT11 and WNT receptors in prostate cancer cell lines (**a**) Q-PCR analysis showing expression levels of the indicated genes, normalized to 36B4, in LNCaP cells, CSS (LNCaP cells that were hormone-depleted by culture in medium containing 5% charcoal-stripped serum for 3 or 6 days), C4-2B, PC-3M and VCaP cells. PC-3M or VCaP were used for normalization in cases where a gene was undetectable (UND) in C4-2B cells (see Supplementary Table 6); error bars show SD for three independent experiments. (**b**) FZD8 mRNA expression levels (ΔCt values - low ΔCt corresponds to high expression) in prostate cancer cell lines ordered from low to high capacity for metastasis.

Supplementary Figure 2. Wnt receptor mRNA expression in prostate cancer databases

a

	Dataset	Comparison	Sample number	P-value	Fold change
**FZD4**	Lapointe Prostate	Carcinoma vs Normal	112	1,61E-11	1,724
	Varambally	Carcinoma vs Normal	13	1,03E-04	1,761
	Tomlins	PIN vs Normal	36	1,63E-04	3,917
	Luo	Carcinoma vs Normal	30	0,023	1,523
**FZD8**	Tomlins	Carcinoma vs Normal	52	3,67E-06	5,057
		PIN vs Normal	35	2,26E-04	2,462
	Varambally	Carcinoma vs Normal	13	0,002	2,803
	Arredouani	Carcinoma vs Normal	21	0,001	1,811
	Grasso	Carcinoma vs Normal	85	1,47E-05	1,717
**PTK7**	Tomlins	Carcinoma vs Normal	47	0,001	2,208
		PIN vs Normal	29	4,02E-04	3,466

b

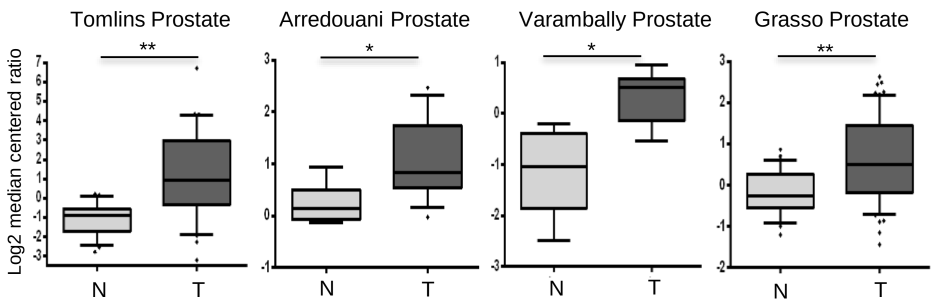

c

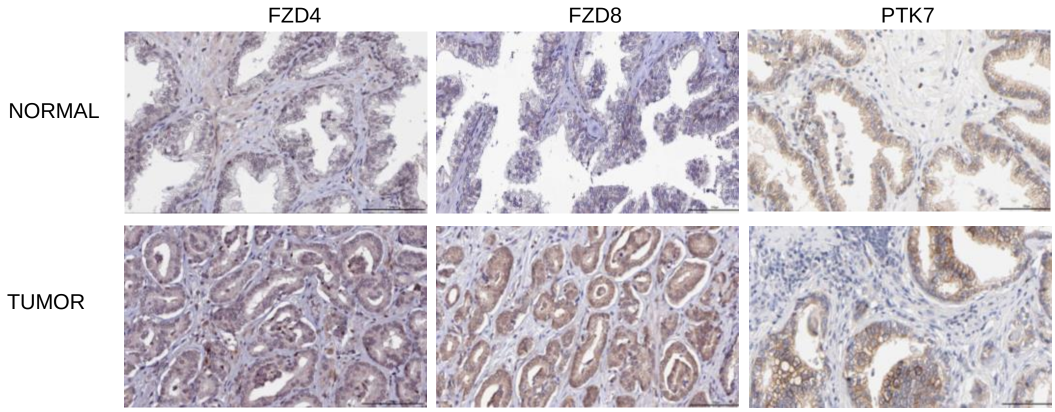

d

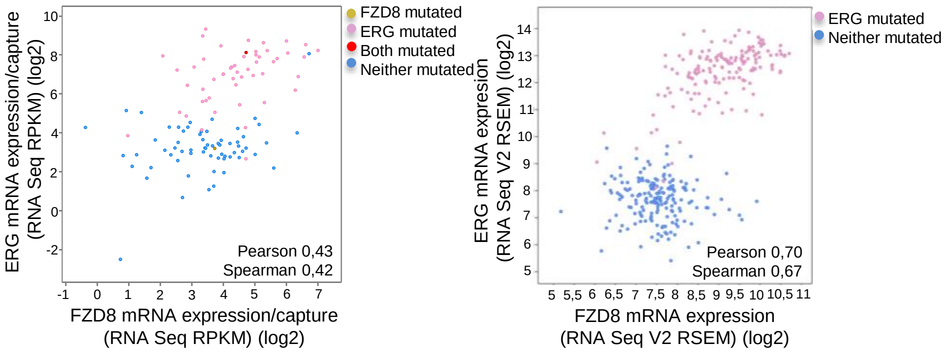

e

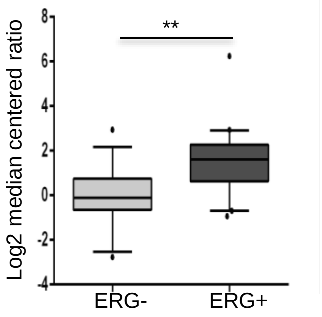

Supplementary Figure 2. Wnt receptor mRNA expression in prostate cancer databases (**a**) FZD4, FZD8 and PTK7 expression in prostate cancer datasets; only datasets with $p < 0.05$ and fold-change > 1.5 were considered (**b**) Analysis of FZD8 expression in four different datasets taken from Oncomine™ (www.Oncomine.org): Tomlins (22 prostate gland and 30 prostate carcinomas), Arredouani (8 prostate gland and 13 prostate carcinomas), Varambally (6 prostate gland and 7 carcinomas) and Grasso (26 prostate gland and 59 carcinomas); prostate gland was defined as normal (N) and prostate carcinoma as tumor (T); only datasets with $p < 0.05$ and fold-change > 1.5 were considered. (**c**) Immunohistochemical detection of FZD_4, FZD_8 and PTK7 in sections of normal prostate and prostate cancer; images were collected from www.Proteinatlas.org. (**d**) Correlation of FZD8 and ERG mRNA expression in prostate cancer datasets from cBioPortal (www.cBioPortal.org), showing plots from the SU2C/PCF Dream Team Metastatic Prostate Cancer dataset (Robinson et al., 2015) (left) and the TGCA prostate cancer dataset (Cancer Genome Atlas Research Network, Cell, 2015) (right) with Pearson and Spearman coefficients. (**e**) FZD8 expression in prostate tumors with (ERG+) and without (ERG-) ERG rearrangement from the Grasso dataset, taken from Oncomine™).

Supplementary Figure 3. Colocalization analysis of Wnt-11 with FZD family members

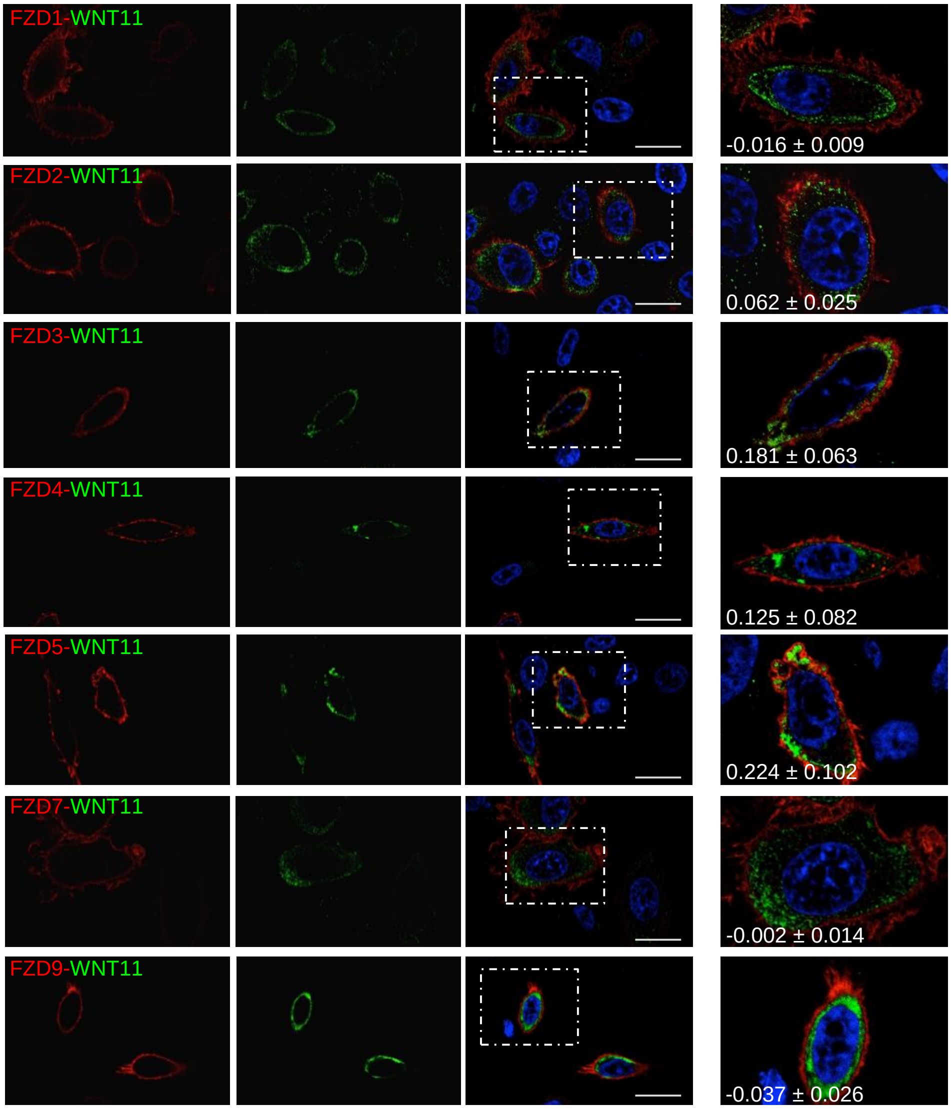

Supplementary Figure 3. Colocalization analysis of Wnt-11 with FZD family members
Confocal microscopy analysis of PC-3M cells transfected with the indicated 1D4-tagged FZD family members (red) and Wnt-11 (green) for 24 h; low magnification images of single color and dual channels and high magnification images of single cells are shown; anti-1D4 and goat anti-Wnt-11 (R&D) were used to detect FZDs and Wnt-11, respectively; blue staining shows cell nuclei (DAPI), images are representative of three independent experiments, scale bars 25 µm. Quantification of colocalization was determined by ImageJ (see Methods) using 10 cells per experiment. Numbers in the images correspond to Pearson correlation coefficient as determined by average and standard desviation.

Supplementary Figure 4. FZD8 silencing effects are specific

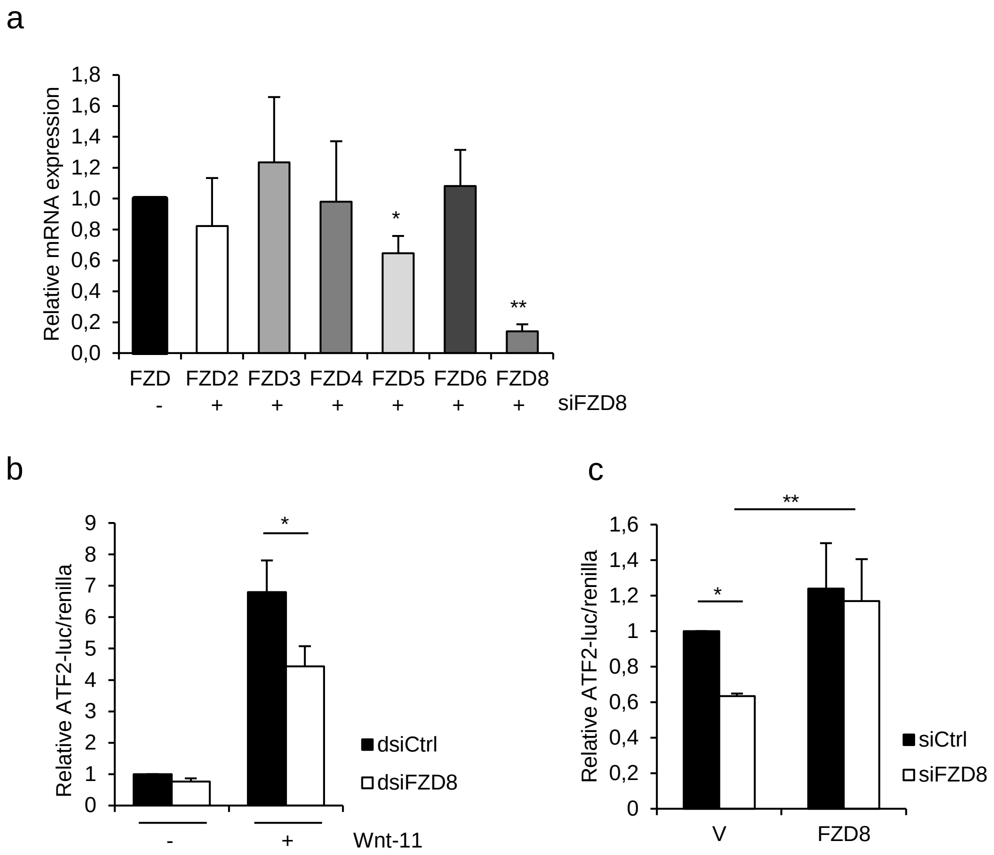

Supplementary Figure 4. FZD8 silencing effects are specific (**a**) Q-PCR analysis showing relative expression levels of the indicated genes, normalized to 36B4, in PC-3M cells transfected with control and FZD8 siRNAs; error bars show SD for four independent experiments (*P<0.05, **P<0,001 by ANOVA with Tukey post-hoc test). (**b**) Relative ATF2 luciferase/renilla activity in PC-3M cells transfected with Wnt-11 and control and FZD8 dsiRNAs and Wnt-11 transfection; error bars show SD for three independent experiments (*P<0.05 by ANOVA with Tukey post-hoc test). (**c**) Relative ATF2 luciferase/renilla activity in PC-3M cells transfected with Wnt-11 and control or FZD8 siRNAs and empty vector (V) or FZD8 plasmid; error bars show SD for four independent experiments (*P<0.05, **P<0,001 by ANOVA with Tukey post-hoc test).

Supplementary Figure 5. FZD8 is required for prostate cancer cell migration and invasion

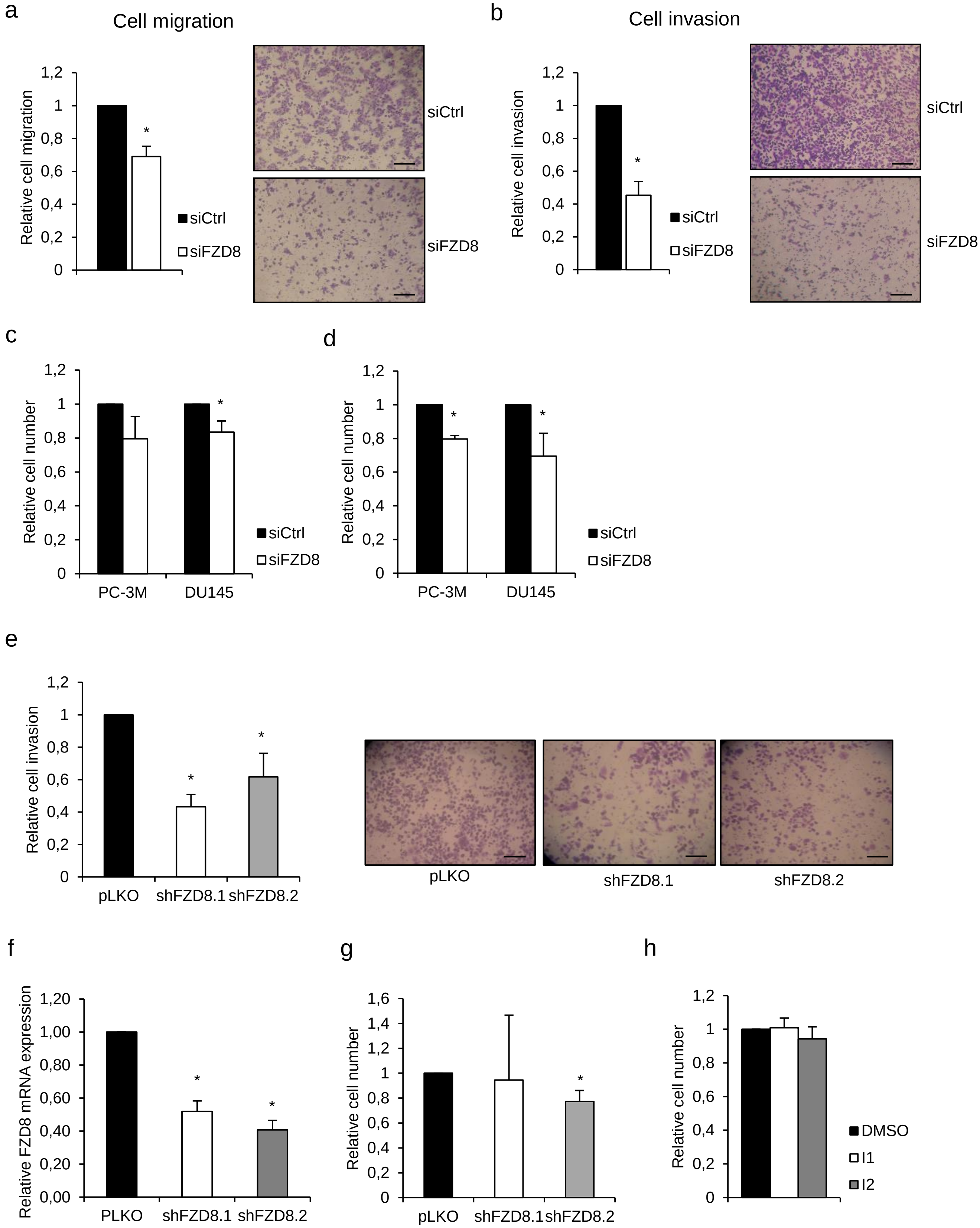

Supplementary Figure 5. FZD8 is required for prostate cancer cell migration and invasion (**a**) and (**b**) Migration and invasion assays, respectively for DU145 cells transfected with control (siCtrl) and FZD8 siRNAs; values are relative to siCtrl and normalized to viable cell number of transfected cells plated in parallel (Supplementary Fig. 5c and d, respectively); error bars show SD for three or four independent experiments, respectively (*P<0.05 by Student´s t test). Representative images are on the right, scale bar 100 µm. (**c**) Cell number of PC-3M and DU145 cells transfected with control and FZD8 siRNAs; error bars show SD of at least three independent experiments (*P<0.05 by Student´s t test). These data were used to normalize numbers of migrating cells in Fig. 2a and Supplementary Fig. 5a. (**d**) Cell number of PC-3M and DU145 cells transfected with control and FZD8 siRNAs; error bars show SD of at least three independent experiments (*P<0.05 by Student´s t test). These data were used to normalize numbers of invading cells in Fig. 2b and Supplementary Fig. 5b. (**e**) Invasion assays using PC-3M cells stably silenced for FZD8 using lentiviruses expressing two different FZD8 shRNAs (shFZD8.1 and shFZD8.2), values are relative to pLKO and normalized to numbers of viable cells plated in parallel (Supplementary Fig. 5g). Error bars show SD for three independent experiments (*P<0.05 by ANOVA with Tukey post-hoc test). Representative images of invaded cells are on the right, scale bar 100 µm. (**f**) Relative mRNA expression levels of FDZ8 measured by q-PCR in shFZD8 PC-3M cells; error bars represent SD of three independent experiments (*P<0.05 by ANOVA with Tukey post-hoc test). (**g**) Cell number of stable PC-3M cells expressing the indicated shRNAs; error bars show SD of three independent experiments (*P<0.05 by ANOVA with Tukey post-hoc test). These data was used to normalize numbers of invading cells in Supplementary Fig. 5e. (**h**) Cell number of PC-3M cells treated with DMSO, inhibitor 1 (I1) and inhibitor 2 (I2); error bars show SD for four independent experiments (*P<0.05 by ANOVA with Tukey post-hoc test). These data were used for normalization the migration experiments in Fig. 2c.

Supplementary Figure 6. FZD8 is required for expression of epithelial-mesenchymal transition (EMT) genes.

a

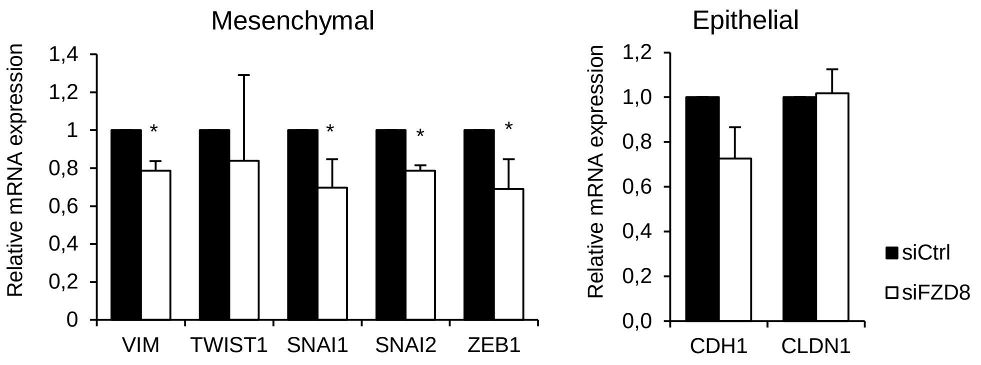

b

		Mesenchymal				Epithelial	
	Correlation coefficient	SNAI1	SNAI3	TWIST1	TWIST2	CDH1	CTNNB1
**FZD8**	**Pearson**	0,57	0,71	0,64	0,6	-0,33	-0,63
	**Spearman**	0,58	0,7	0,66	0,63	-0,35	-0,64
**WNT11**	**Pearson**	0,47	0,57	0,36	0,49	-0,39	-0,52
	**Spearman**	0,57	0,71	0,42	0,61	-0,46	-0,62

c

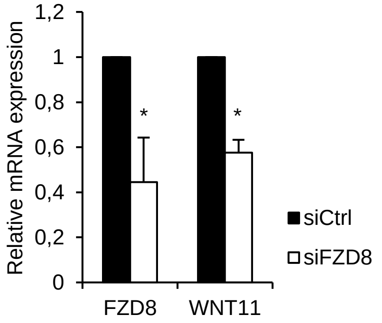

d

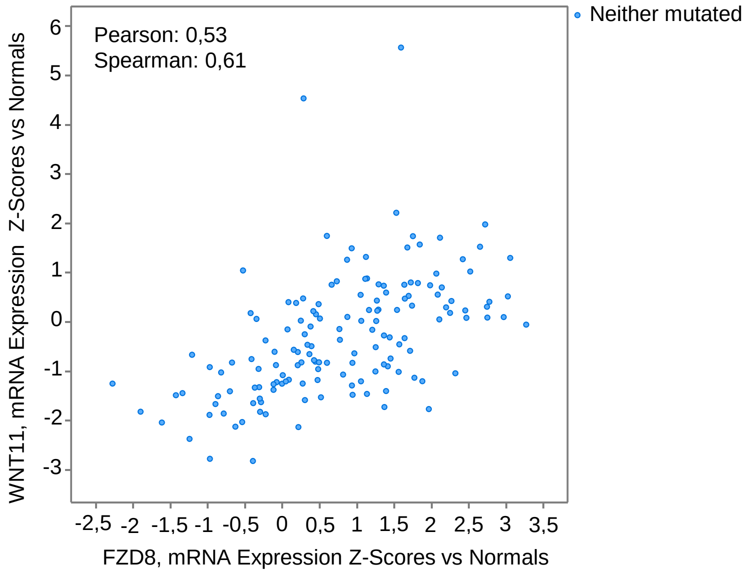

Supplementary Figure 6. FZD8 is required for expression of epithelial-mesenchymal transition (EMT) genes (**a**) Q-PCR analysis showing expression levels of the indicated EMT genes, normalized to 36B4, in DU145 cells transfected with control (siCtrl) or FZD8 siRNAs; error bars show SD for four independent experiments (*P<0.05 by Student´s t test). (**b**) Correlation analysis of FZD8 and WNT11 mRNA expression with the indicated mesenchymal and epithelial genes, as determined using the MSKCC dataset and calculated using cBioPortal; Pearson and Spearman coefficients were used to measure correlation. (**c**) Relative expression levels of FDZ8 and WNT11 measured by q-PCR in FZD8-silenced DU145 cells; error bars represent SD of four independent experiments (*P<0.05 by Student´s t test). (**d**) Correlation plot for FZD8 and WNT11 mRNA expression in the MSKCC prostate cancer dataset, generated using cBioPortal. Pearson and Spearman coefficients were used to measure correlation.

Supplementary Figure 7. FZD8 is required for prostate cancer cell invasion in organotypic 3D cultures

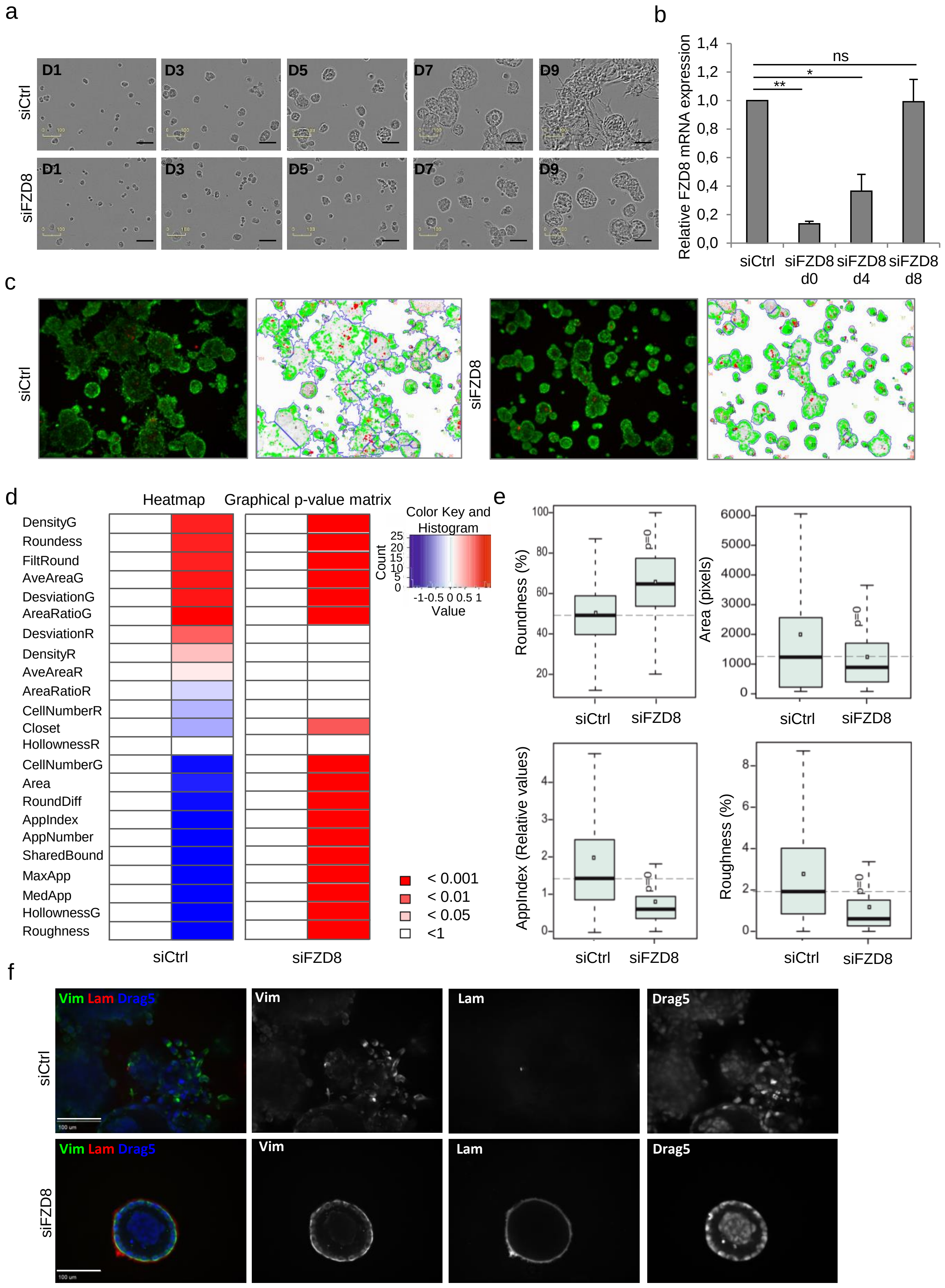

Supplementary Figure 7. FZD8 is required for PC-3 prostate cancer cell invasion in organotypic 3D cultures (**a**) Representative images from live-cell imaging of 3D cultures of control and FZD8-silenced PC-3 cells at days 1 to 9; scale bar 100 µm. (**b**) FZD8 expression levels in siRNA-transfected cells at days 0, 4 and 8 of 3D culture. Error bars indicate SD from three independent experiments (*P< 0.05, **P<0.001 by ANOVA with Tukey post-hoc test, ns indicate non significant). (**c**) Representative segmentation of live-cell spinning disk confocal images of FZD8-silenced PC-3 cell organoids cultured in 3D for 9 days. Organoids were segmented and analyzed by AMIDA; apoptotic cells are in red (ethidium homodimer) and live cells in green (calcein). (**d**) Heatmaps and graphical p-value matrix of morphometric parameters measured by AMIDA and found to be altered by FZD8 silencing (red, increased and blue decreased). P values displayed in the figure are Bonferroni-corrected from t-tests, comparing siFZD8 and siCtrl. (**e**) Box and whisker plots of selected parameters from heatmaps; p=0 indicates p-value <0.001. For explanation of the morphometric parameters, see Supplementary Table 6. (**f**) Confocal microscopy analysis of organoids derived from siCtrl and FZD8 silenced PC-3M cells at day 9 of growth in 3D culture; immunostaining for vimentin (Vim) is shown in green and for laminin-a1 (Lam) in red, blue staining shows cell nuclei (Draq5), scale bar 100 µm.

Supplementary Figure 8. FZD8 and WNT11 involvement in prostate cancer relapse

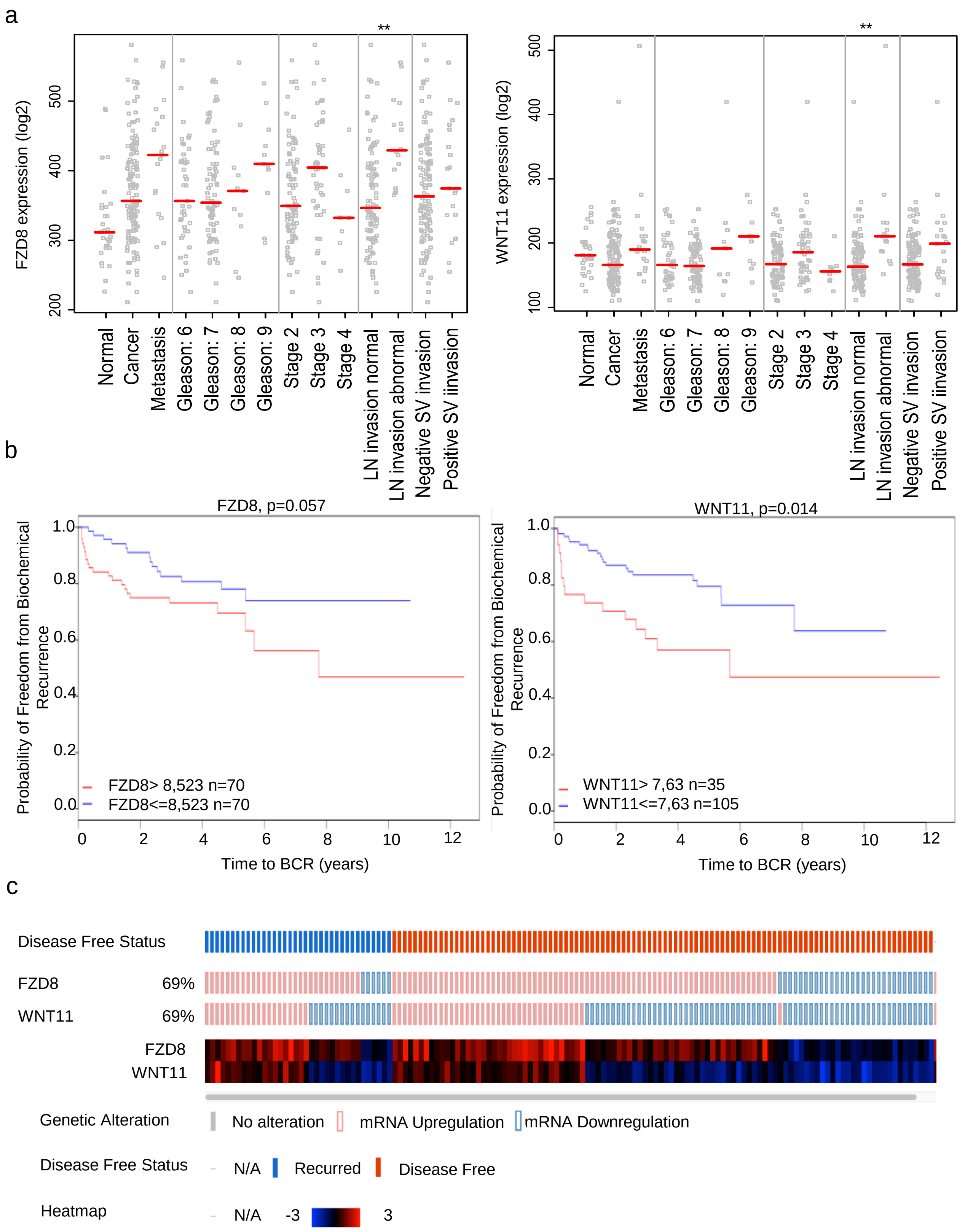

Supplementary Figure 8. Correlation of FZD8 and WNT11 with prostate cancer relapse (**a**) Bioinformatics analysis of FZD8 (left panel) and WNT11 (right panel) expression in prostate cancer using the MSKCC dataset (**P<0,001 by U-Mann Whitney test). (**b**) Recurrence analysis based on FZD8 and WNT11 expression in the MSKCC dataset, determined using CamcAPP (*P<0,005 by Recursive partitioning). (**c**) Comparison of tumors with changes in FZD8 and WNT11 expression (up, red; down, blue) and disease-free status of patients, from cBioPortal.

Supplementary Figure 9. FZD8 is required for TGF-β signaling

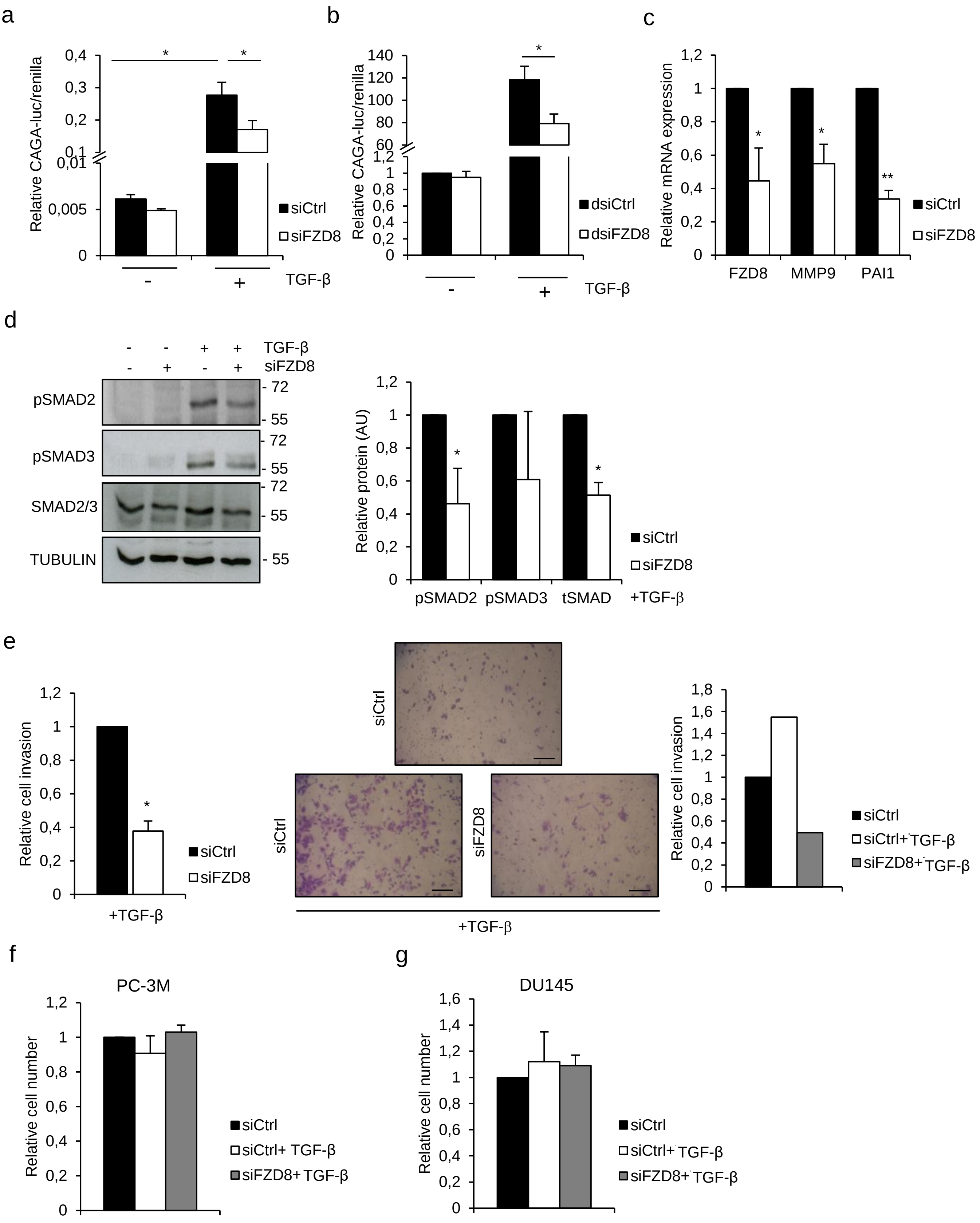

Supplementary Figure 9. FZD8 regulates TGF-β signaling (**a**) Relative CAGA12-luciferase/renilla activity in DU145 cells transfected with control or FZD8 siRNAs and treated -/+ 0.1 ng ml^{-1} TGF-β for 24 h; error bars show SD from three independent experiments (*P<0.05 by ANOVA with Tukey post-hoc test). (**b**) Relative CAGA12-luciferase/renilla activity in PC-3M cells transfected with control or dsiFZD8 and treated -/+ 1 ng ml^{-1} TGF-β for 24 h; error bars show SD from three independent experiments (*P<0.05 by ANOVA with Tukey post-hoc test). (**c**) Q-PCR analysis of the indicated genes in DU145 cells transfected with control or FZD8 siRNAs; error bars show SD for four independent experiment (*P<0.05, **P<0,001 by Student´s t-test). (**d**) Western blots of extracts from DU145 cells transfected with control or FZD8 siRNAs and treated with 1 ng ml^{-1} TGF-β for 30 min were probed for pSMAD2, pSMAD3, SMAD2/3 and β-tubulin as a loading control; graph on the right shows relative levels of expression, as determined by densitometry analysis of bands from TGF-β-treated extracts, normalized to β-tubulin and relative to control siRNA, from three independent experiments (*P<0.05 by Student´ s t-test). (**e**) Invasion assays of control and FZD8-silenced DU145 cells treated with 5 ng ml^{-1} of TGF-β for 48h; error bars represent SD of three independent experiments Left: relative cell invasion normalized to siCtrl in the presence of TGF-β, taking into account effects of silencing on cell number (Supplementary Fig. 9g). Middle: representative pictures of invaded cells, scale bar 100 µm. Right: representative experiment. (**f**) and (**g**) Cell number of PC-3M and DU145 cells, respectively transfected with control and FZD8 siRNAs and treated with 5 ng ml^{-1} of TGF-β for 48h; error bars show SD of three independent experiments (*P<0.05 by ANOVA with Tukey post-hoc test). These data were used to normalize numbers of invading cells in Fig. 6d and Supplementary Fig. 9e, respectively.

Supplementary Figure 10. TGF-β effects on expression of EMT-related genes

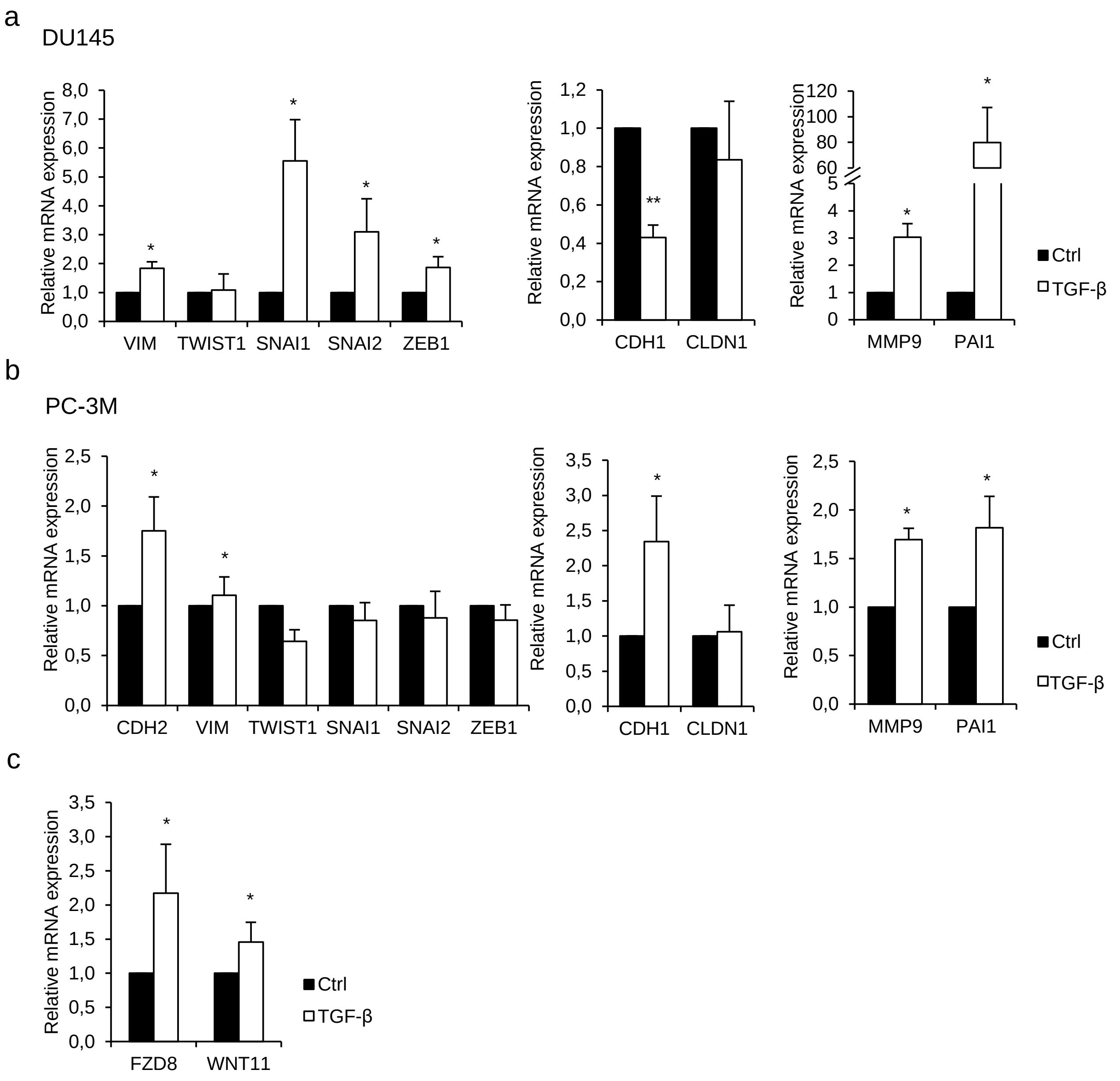

Supplementary Figure 10. TGF-β effects on expression of EMT-related genes (**a**) and (**b**) Q-PCR analysis of the indicated genes in DU145 and PC-3M cells, respectively treated -/+ 1 ng ml^{-1} TGF-β for 24 h; error bars show SD for four independent experiments (*P<0.05, **P<0,001 by Student´s t-test). (**c**) Q-PCR analysis of the indicated genes in DU145 cells treated -/+ 1 ng ml^{-1} TGF-β for 24 h; error bars show SD for four independent experiments (*P<0.05 by Student´s t-test).

Supplementary Figure 11. Contribution of ATF-2 to TGFβ signaling

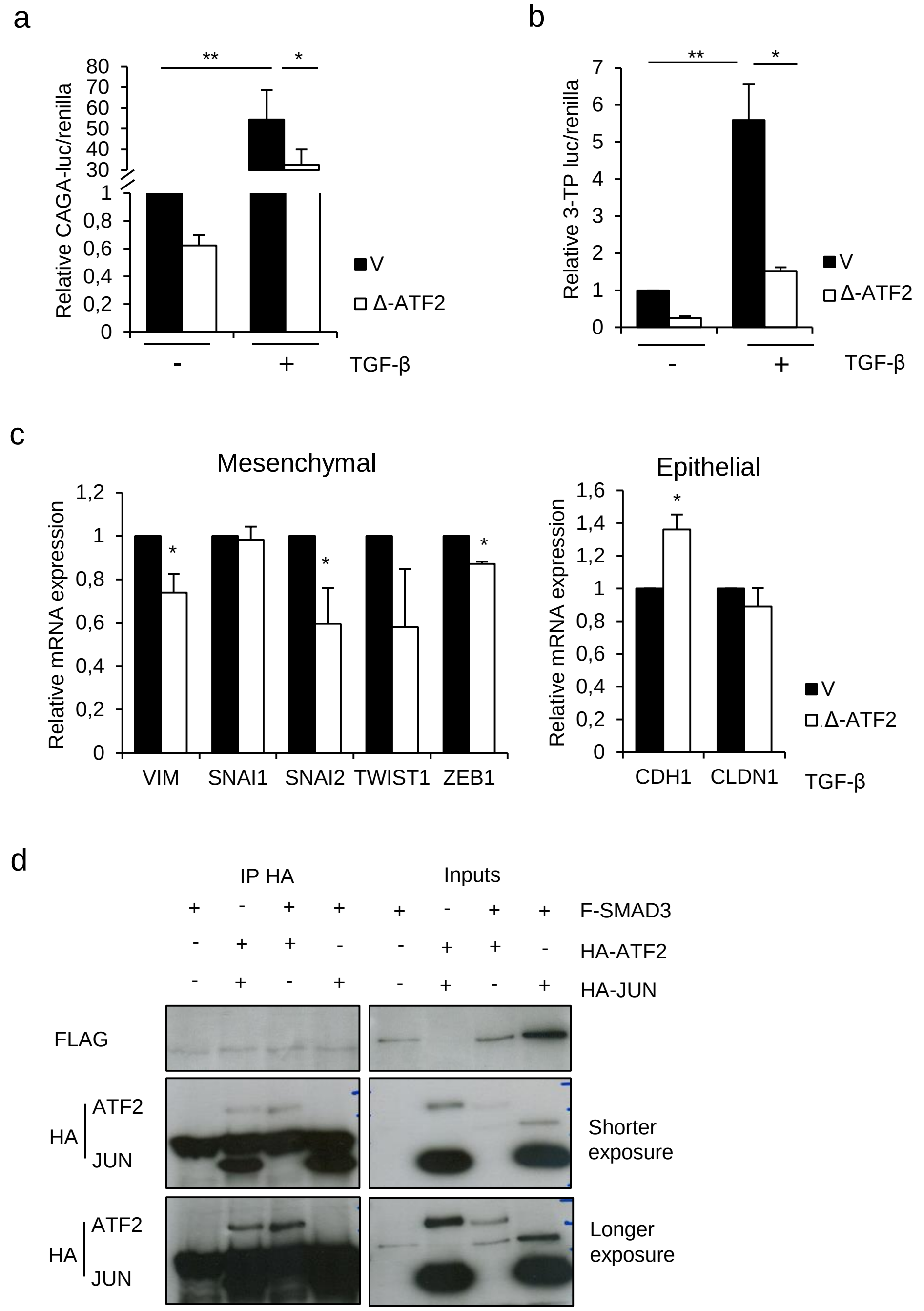

Supplementary Figure 11. Contribution of ATF-2 to TGFβ signaling (**a**) Relative CAGA12-luciferase/renilla activity in PC-3M cells transfected with empty vector (V) or dominant-negative ATF2 (Δ-ATF2) and treated with 1 ng ml^{-1} TGF-β for 24 h; error bars show SD for three independent experiments (*P<0.05, **P<0,001 by ANOVA with Tukey post-hoc test). (**b**) Relative 3TPlux luciferase/renilla activity in PC-3M cells transfected with empty vector (V) or dominant-negative ATF2 (Δ-ATF2) and treated -/+ 1 ng ml^{-1} TGF-β for 24 h; error bars show SD from three independent experiments (*P<0.05, **P<0,001 by ANOVA with Tukey post-hoc test). (**c**) Q-PCR analysis showing mRNA expression of the indicated genes, relative to 36B4, in DU145 cells transfected with empty vector (V) or Δ-ATF2 and treated with 1 ng ml^{-1} TGF-β for 24 h; error bars show SD for four independent experiments (*P<0.05 by Student´s t-test). (**d**) Western blots of anti-HA immunoprecipitates (IP) and extracts (inputs) from PC-3M cells transfected for 24h with HA-tagged ATF2 and c-JUN, and SMAD3 plasmids were probed for ATF2 and c-JUN (HA) and SMAD3 (Flag); blots are representative of two independent experiments.

Supplementary Figure 12. Uncropped scans of Western Blots

Figure 2 g

IP 1D4

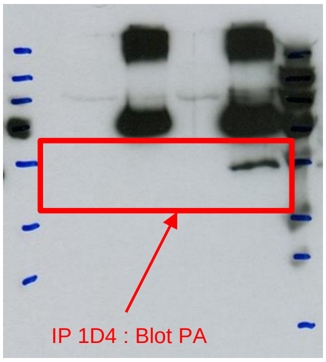

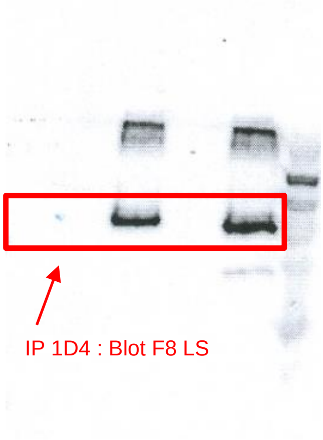

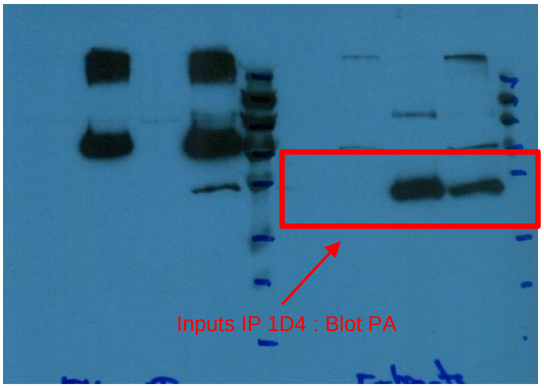

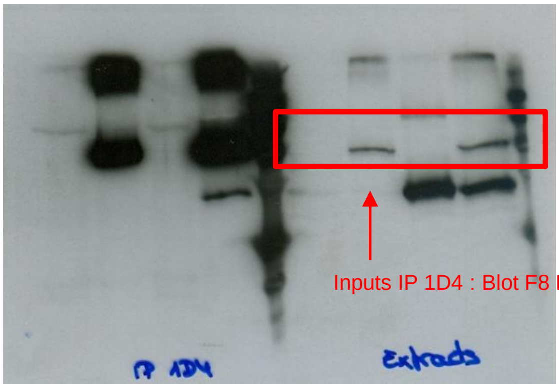

IP PA

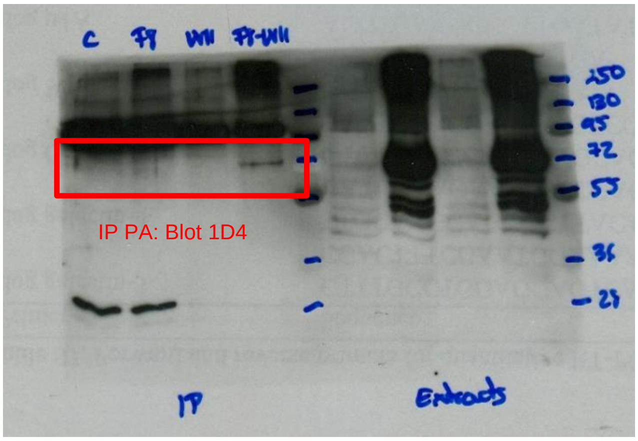

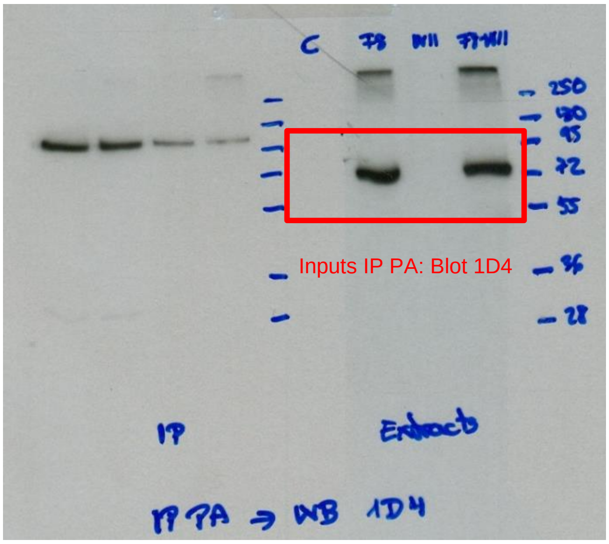

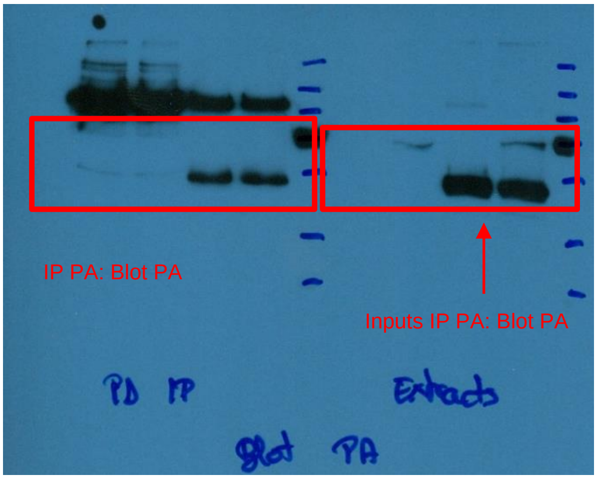

Figure 3e

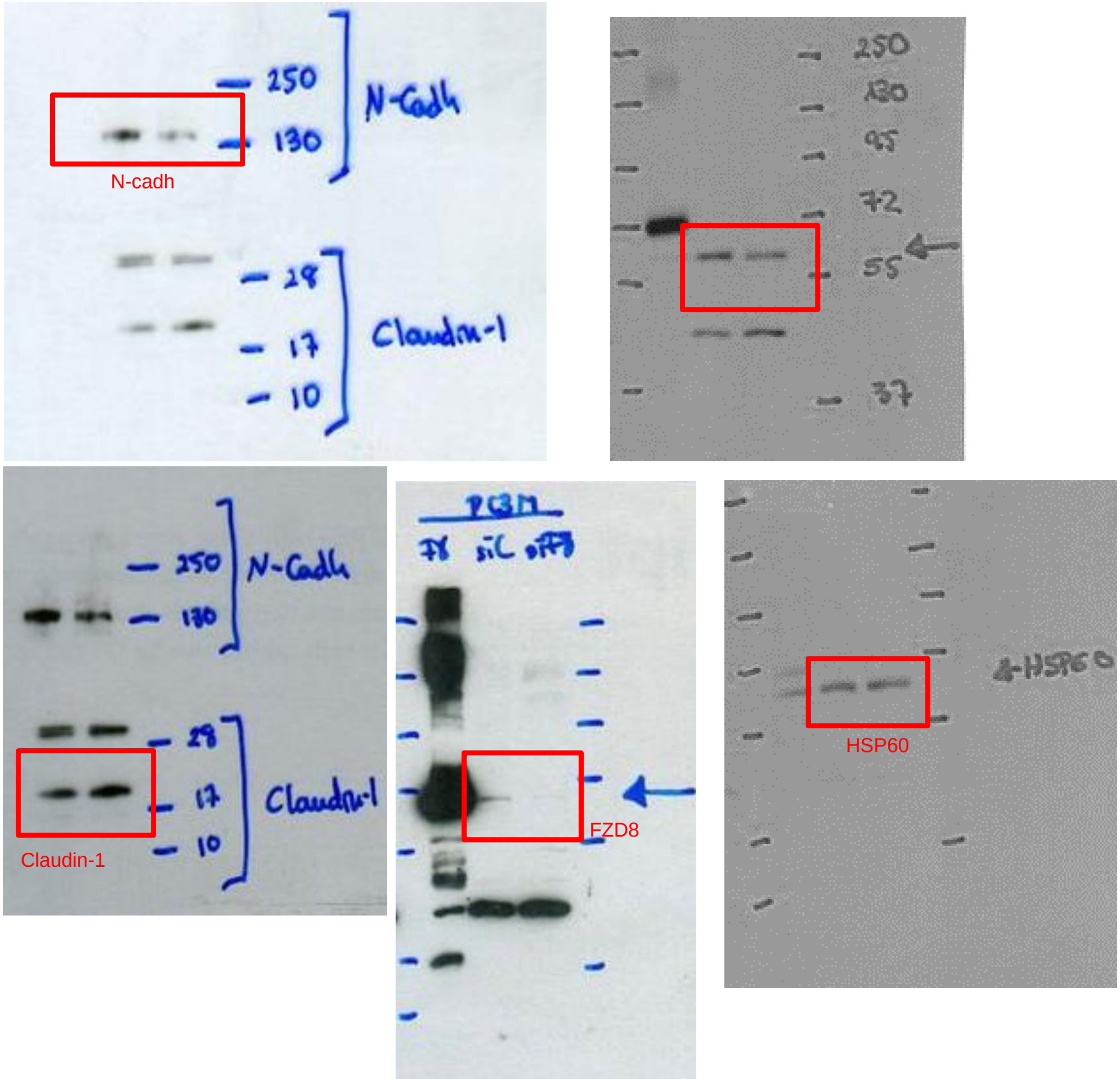

Figure 7c

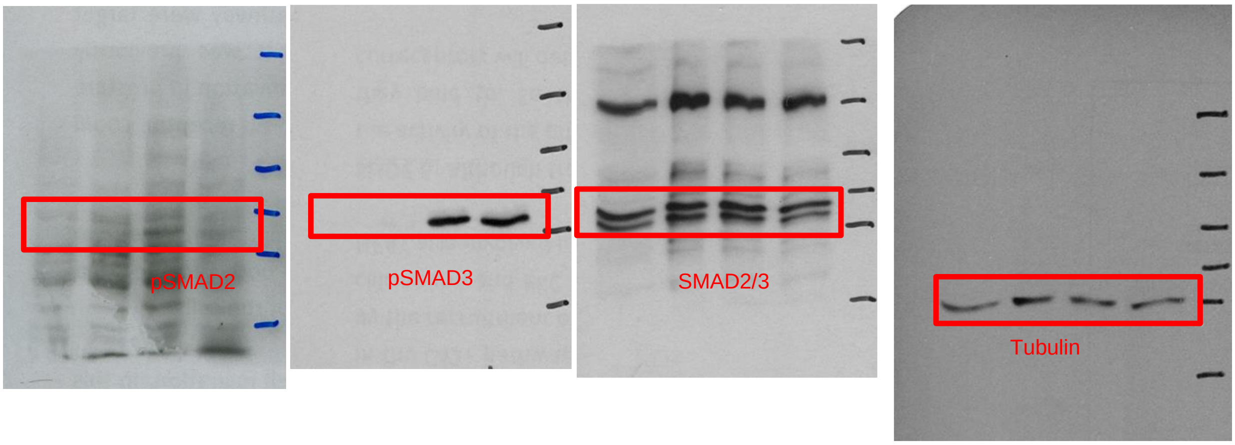

Figure 8a

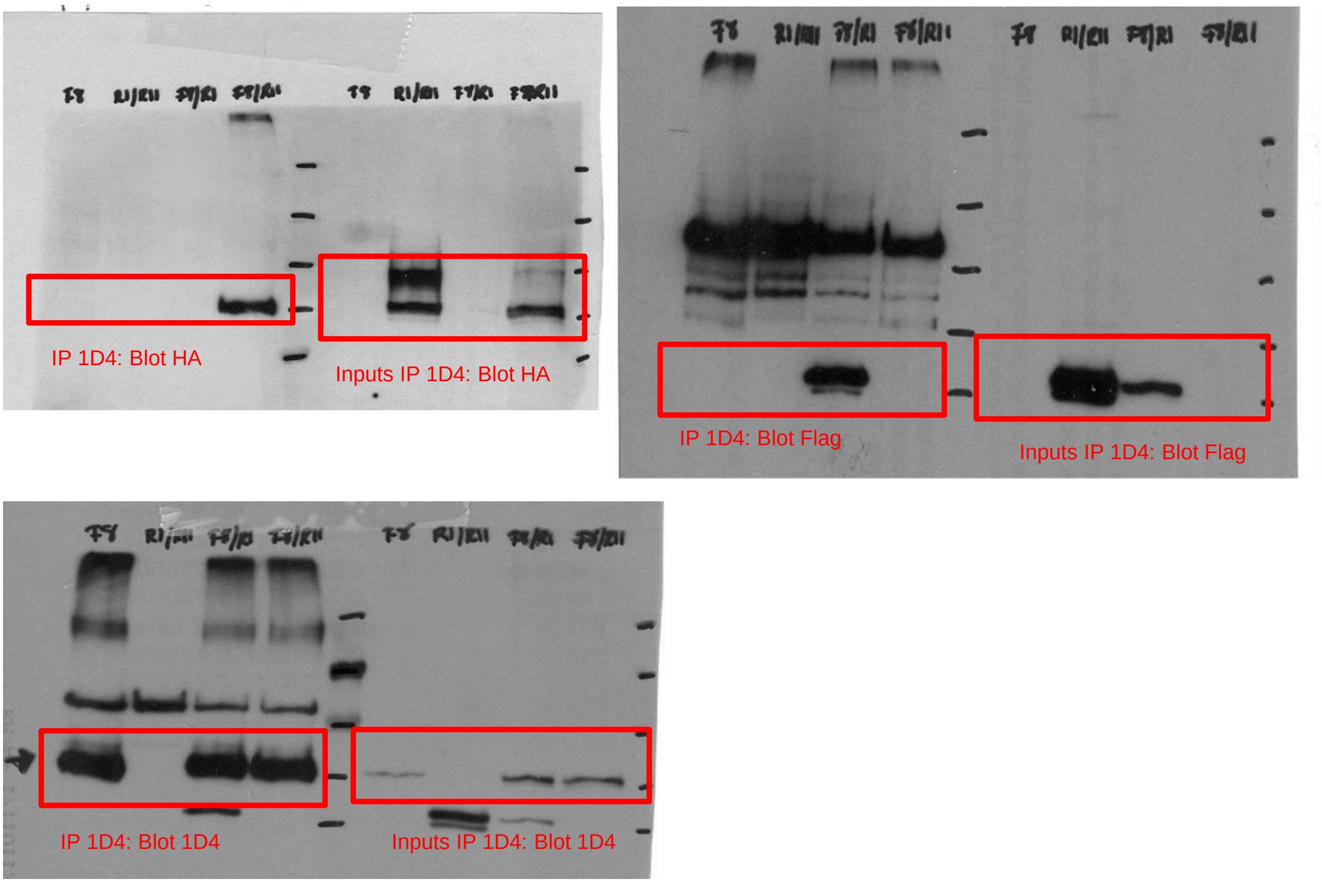
IP 1D4: Blot HA
Inputs IP 1D4: Blot HA
IP 1D4: Blot Flag
Inputs IP 1D4: Blot Flag
IP 1D4: Blot 1D4
Inputs IP 1D4: Blot 1D4

Figure 8c

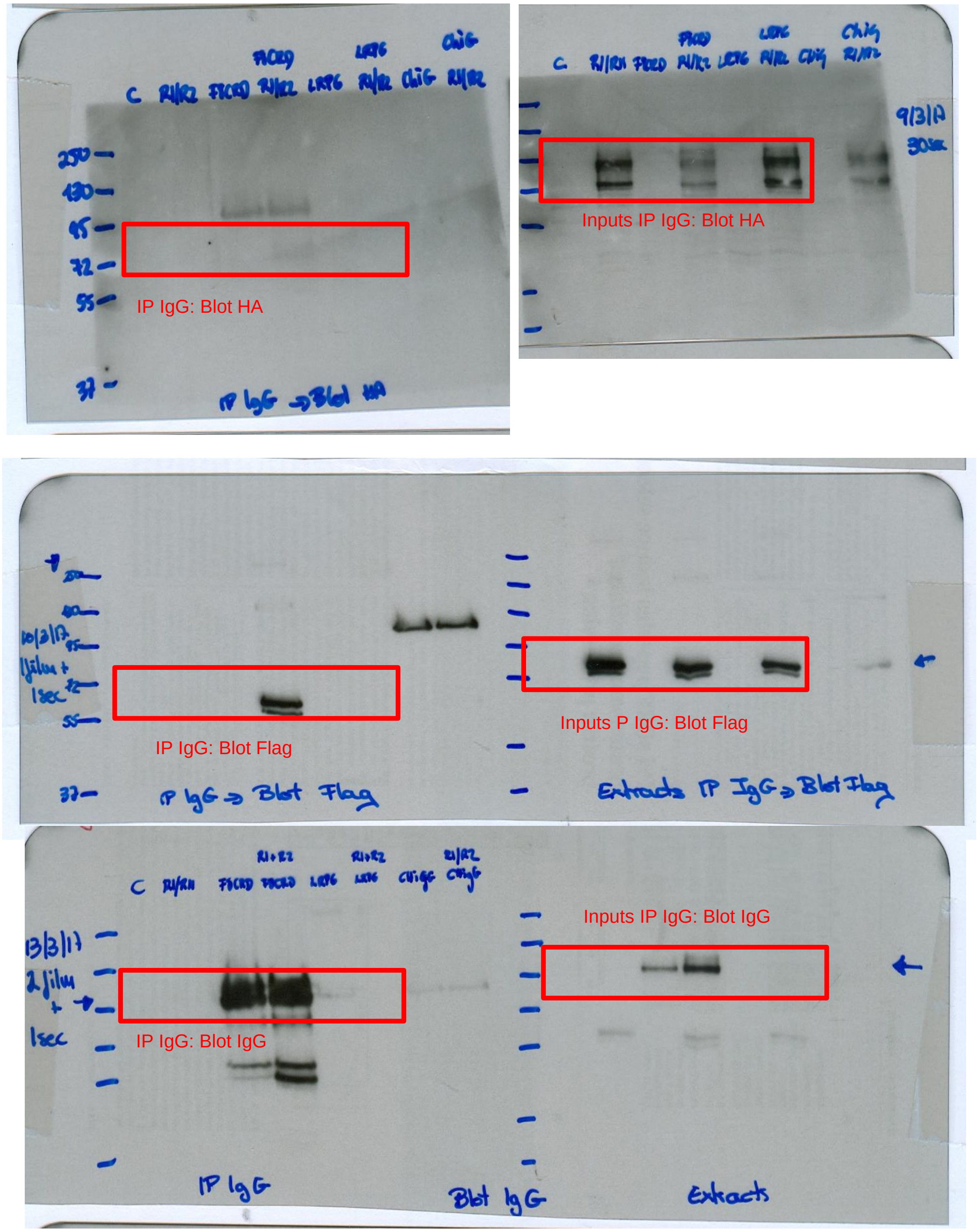
IP IgG: Blot HA
Inputs IP IgG: Blot HA
IP IgG: Blot Flag
Inputs P IgG: Blot Flag
Inputs IP IgG: Blot IgG
IP IgG: Blot IgG

Figure 8c (continuation)

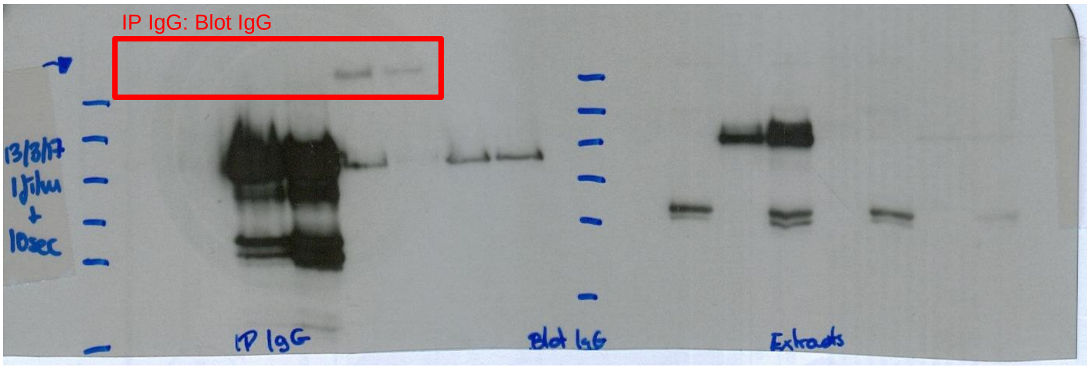

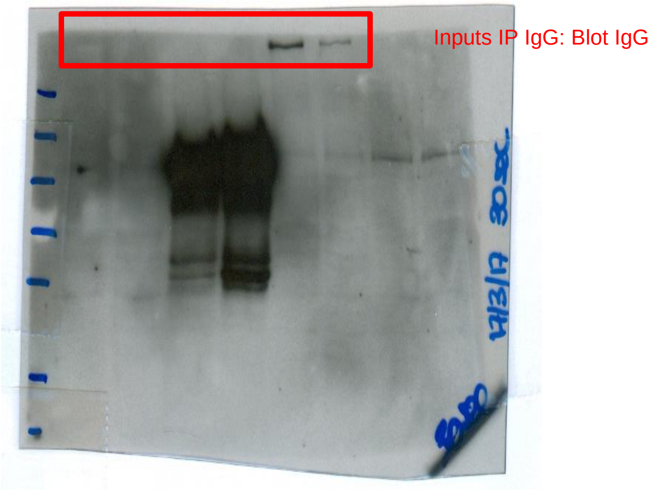

Figure 8d

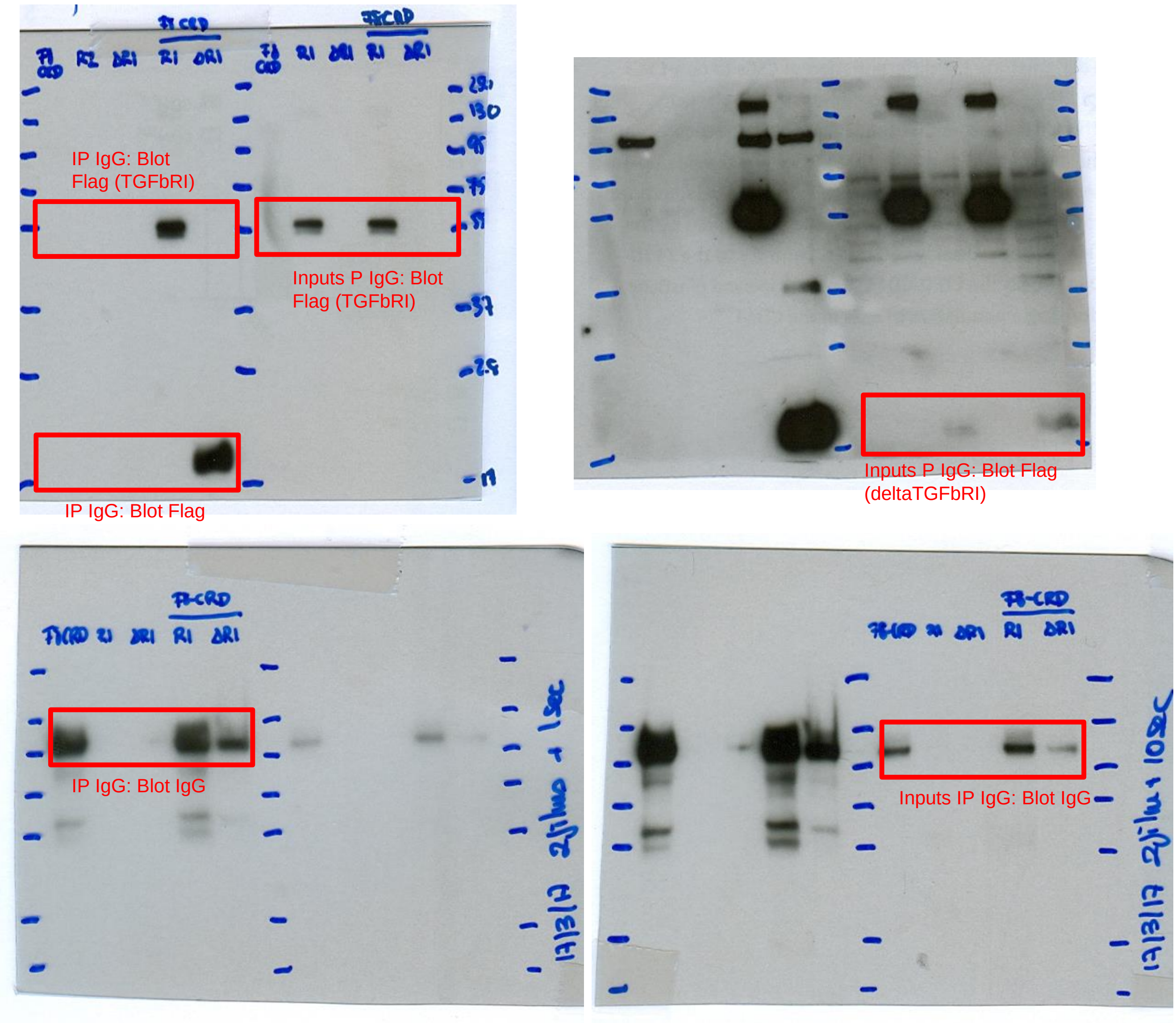
IP IgG: Blot Flag (TGFbRI)
Inputs P IgG: Blot Flag (TGFbRI)
IP IgG: Blot Flag
Inputs P IgG: Blot Flag (deltaTGFbRI)
IP IgG: Blot IgG
Inputs IP IgG: Blot IgG

Figure 8e

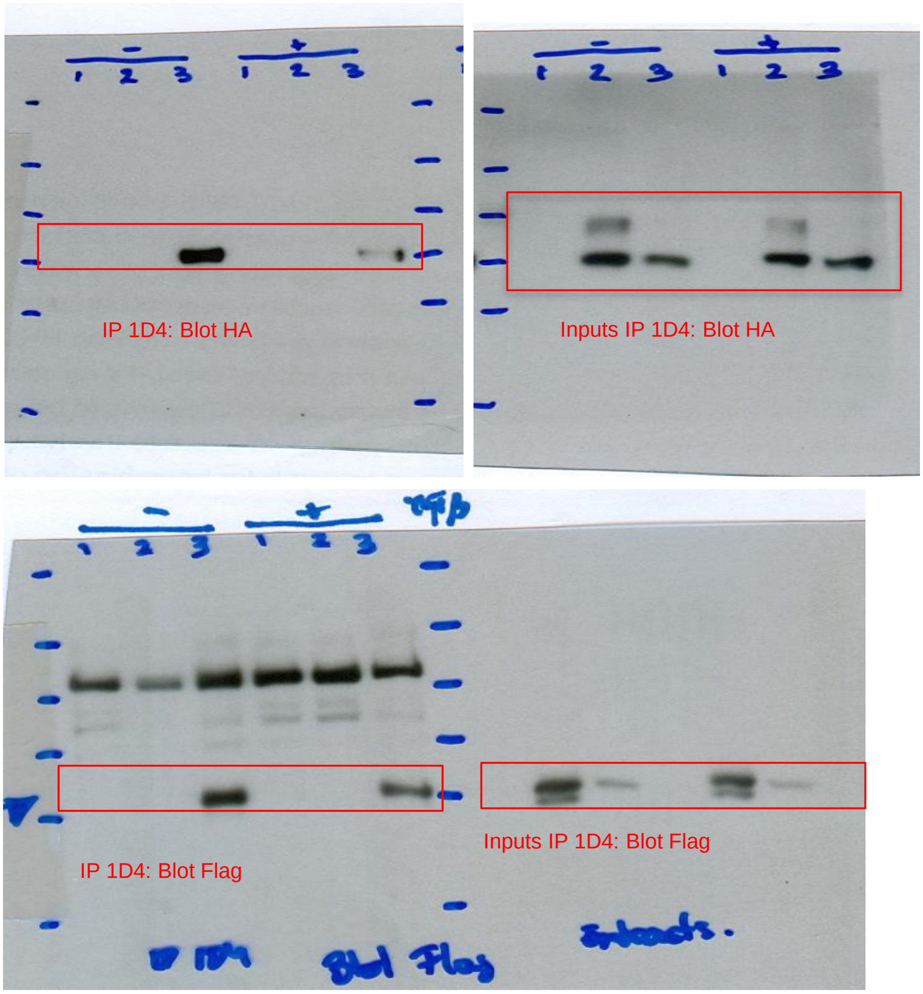
IP 1D4: Blot HA
Inputs IP 1D4: Blot HA
IP 1D4: Blot Flag
Inputs IP 1D4: Blot Flag

Figure 8e (continuation)

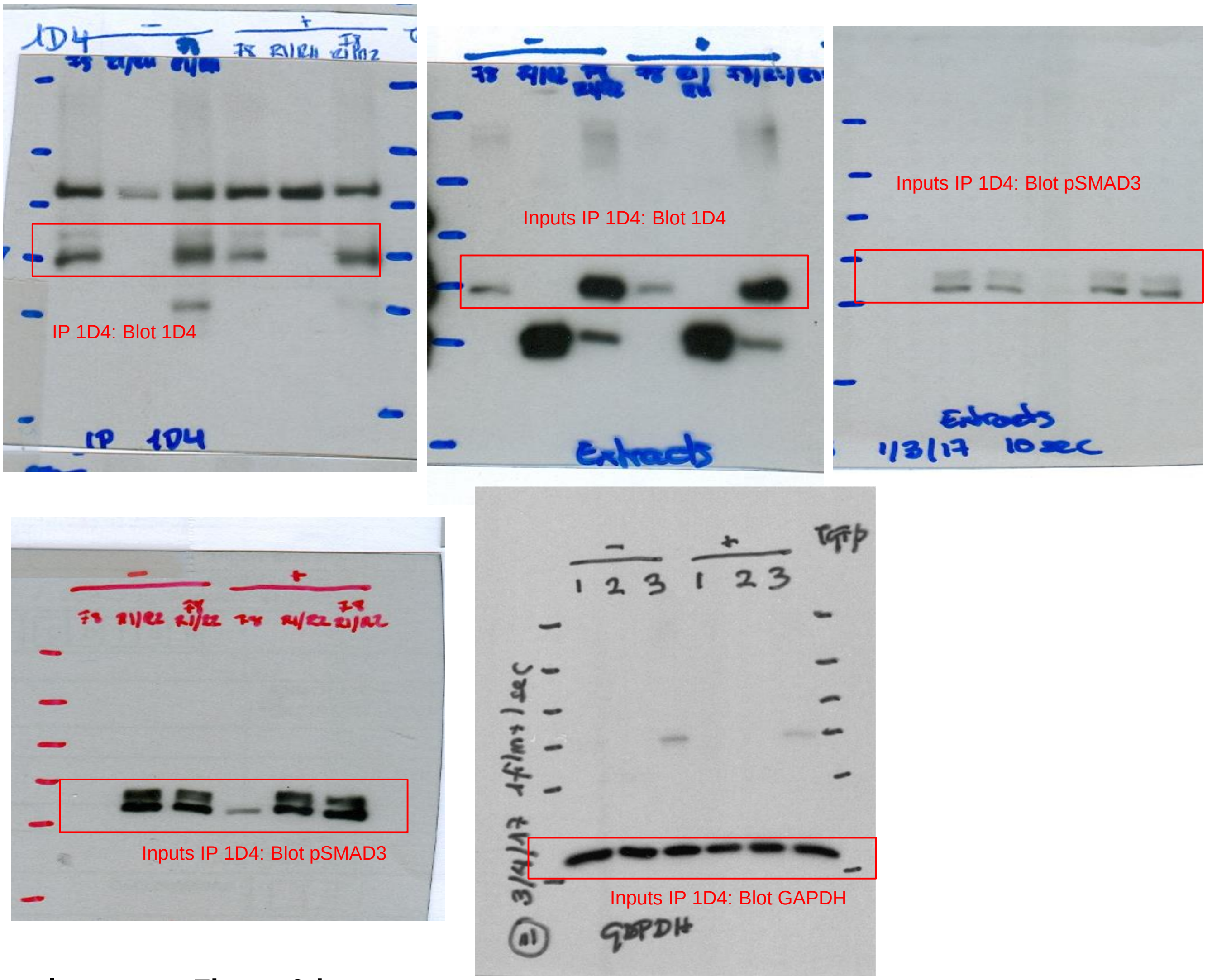

Supplementary Figure 9d

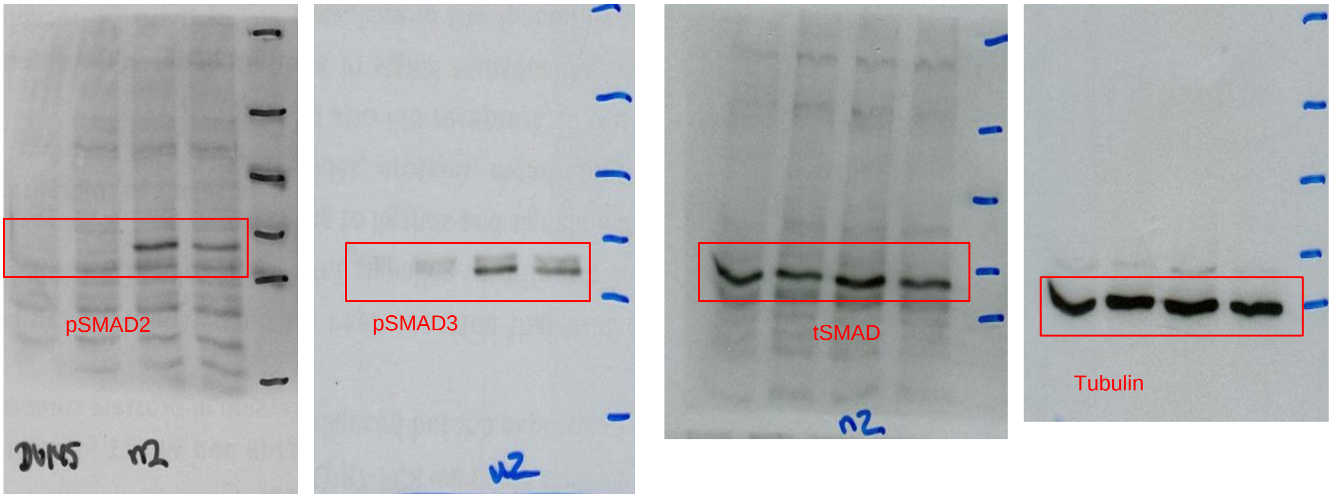

Supplementary Figure 11d

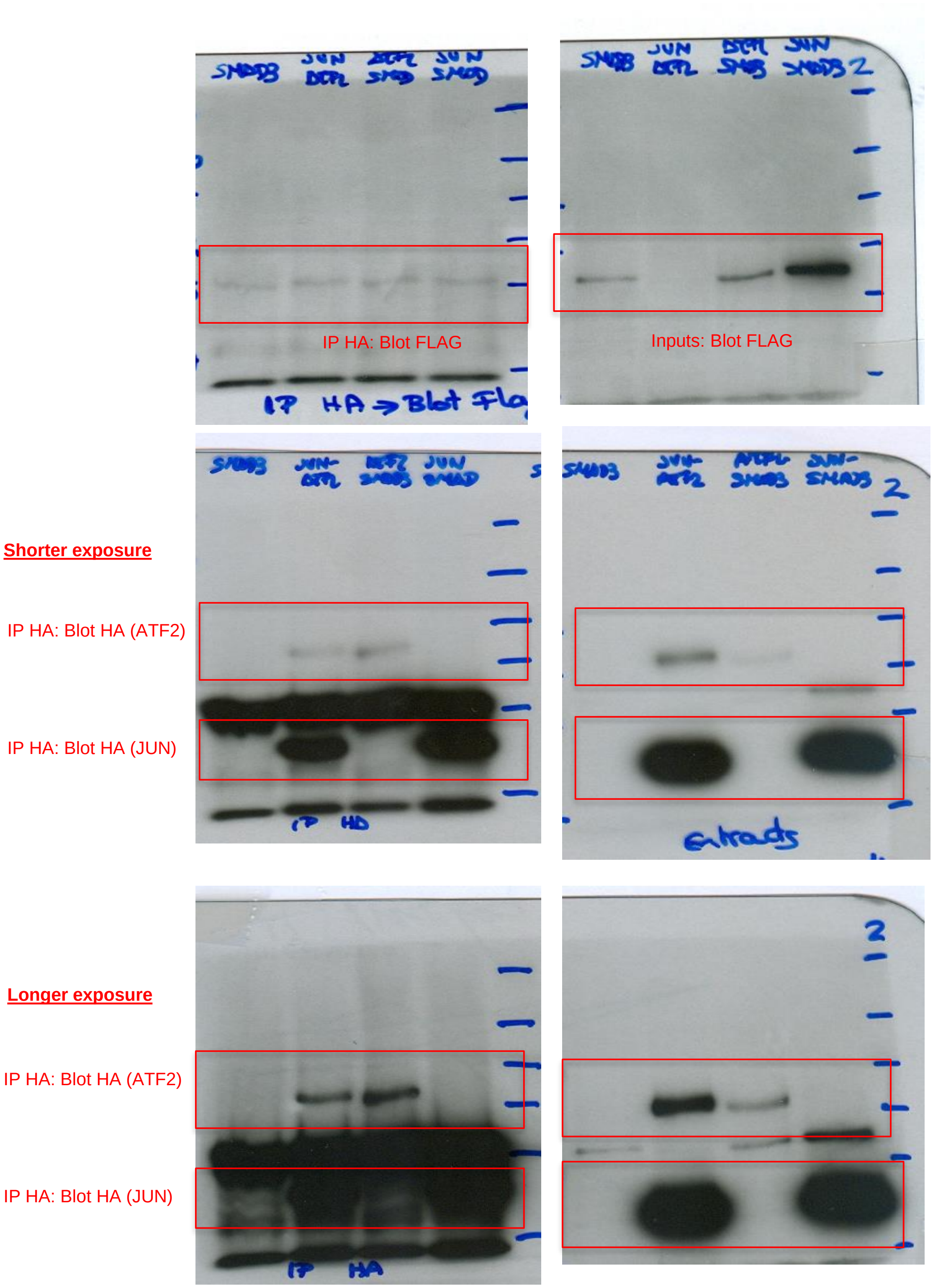

Supplement: Supplementary file 2 — Supplementary Information [file 41467_2018_4042_MOESM2_ESM.pdf]
